# Predicting outcome in patients with moderate to severe traumatic brain injury using electroencephalography

**DOI:** 10.1186/s13054-019-2656-6

**Published:** 2019-12-11

**Authors:** Marjolein E. Haveman, Michel J. A. M. Van Putten, Harold W. Hom, Carin J. Eertman-Meyer, Albertus Beishuizen, Marleen C. Tjepkema-Cloostermans

**Affiliations:** 1 Clinical Neurophysiology Group, University of Twente, Drienerlolaan 5, 7522 NB Enschede, the Netherlands; 2 Department of Neurology and Clinical Neurophysiology (C2), Medisch Spectrum Twente, Koningsplein 1, 7512 KZ Enschede, the Netherlands; 3 Intensive Care Center, Medisch Spectrum Twente, Koningsplein 1, 7512 KZ Enschede, the Netherlands

**Keywords:** Traumatic brain injury, EEG, Prognosis, Random forest, ICU

## Abstract

**Background:**

Better outcome prediction could assist in reliable quantification and classification of traumatic brain injury (TBI) severity to support clinical decision-making. We developed a multifactorial model combining quantitative electroencephalography (qEEG) measurements and clinically relevant parameters as proof of concept for outcome prediction of patients with moderate to severe TBI.

**Methods:**

Continuous EEG measurements were performed during the first 7 days of ICU admission. Patient outcome at 12 months was dichotomized based on the Extended Glasgow Outcome Score (GOSE) as poor (GOSE 1–2) or good (GOSE 3–8). Twenty-three qEEG features were extracted. Prediction models were created using a Random Forest classifier based on qEEG features, age, and mean arterial blood pressure (MAP) at 24, 48, 72, and 96 h after TBI and combinations of two time intervals. After optimization of the models, we added parameters from the International Mission for Prognosis And Clinical Trial Design (IMPACT) predictor, existing of clinical, CT, and laboratory parameters at admission. Furthermore, we compared our best models to the online IMPACT predictor.

**Results:**

Fifty-seven patients with moderate to severe TBI were included and divided into a training set (*n* = 38) and a validation set (*n* = 19). Our best model included eight qEEG parameters and MAP at 72 and 96 h after TBI, age, and nine other IMPACT parameters. This model had high predictive ability for poor outcome on both the training set using leave-one-out (area under the receiver operating characteristic curve (AUC) = 0.94, specificity 100%, sensitivity 75%) and validation set (AUC = 0.81, specificity 75%, sensitivity 100%). The IMPACT predictor independently predicted both groups with an AUC of 0.74 (specificity 81%, sensitivity 65%) and 0.84 (sensitivity 88%, specificity 73%), respectively.

**Conclusions:**

Our study shows the potential of multifactorial Random Forest models using qEEG parameters to predict outcome in patients with moderate to severe TBI.

## Background

There is an ongoing need for continuous, bedside-available, preferably non-invasive tools for reliable prediction of neurological outcome in traumatic brain injury (TBI) patients. A prediction model for patient outcome after TBI might provide realistic information to caregivers to inform relatives about expectations. Besides, better outcome prediction could assist in reliable quantification and classification of TBI severity to support clinical decision-making [1].

Currently, the International Mission for Prognosis And Clinical Trial Design (IMPACT) predictor is the best available predictor for neurological outcome after TBI. This online predictor is based on a large multicenter database including both randomized controlled trials and observational studies in TBI [2]. Linear regression models were created on clinical admission parameters to predict mortality (Glasgow Outcome Scale 1) and unfavorable outcome (Glasgow Outcome Scale 1–3) at 6 months after injury [3]. The model is based on age, motor score, pupillary reaction, CT characteristics, signs of hypoxia, hypotension, glucose, and hemoglobin at admission. However, this prediction model, based on parameters at admission, does not take into account evolvement of secondary injury in TBI patients nor effects from treatment [4].

Electroencephalography (EEG) is a non-invasive bedside-available measure of cortical activity and is particularly useful in sedated or comatose patients [5–7]. Continuous EEG (cEEG) is often part of the multimodal monitoring in TBI patients at the ICU, where it is mainly used for the detection of (non-convulsive or electrographic) seizures [8, 9].

Several attempts have been made to relate EEG features to functional outcome of TBI patients. Worse outcome was associated with lower (regional) EEG power [9], slowing of the EEG [10–13], decrease in alpha power [9–11, 14, 15], lower EEG (alpha) variability [9, 16, 17], and increased coherence [18]. However, most studies used single EEG features and were limited to mild TBI in the subacute phase (days, weeks, or even months) after the moment of injury [19]. A combination of multiple EEG features might be even more relevant for outcome prediction in TBI patients, considering the heterogeneity of the primary injury, additional organ dysfunctions, and evolvement of secondary injury in these patients. Furthermore, computer algorithms can assist in EEG interpretation [6, 20, 21], which decreases the labor intensity and subjectivity.

As proof of concept, we report on a cEEG monitoring tool using multiple quantitative EEG (qEEG) features combined with IMPACT predictors for neurologic outcome prediction in patients with moderate to severe TBI.

## Materials and methods

### Study population

Between 2013 and 2018, two prospective cohort studies were performed in the Medisch Spectrum Twente in which cEEG was recorded in patients with moderate to severe TBI. Inclusion criteria were patients of 18 years and older with moderate to severe TBI (Glasgow Coma Scale ≤ 12 at the trauma location or emergency department) admitted to the ICU with a(n) (expected) stay of more than 24 h. Exclusion criteria were trauma following or combined with severe circulatory failure (cardiac arrest/cerebral hemorrhage), earlier TBI or CVA without full recovery, progressive brain illness (tumor, neurodegenerative disease), or limited life expectancy (< 6 months) prior to TBI. Since the EEG is part of our routine care, the institutional review board waived the need for written informed consent. Verbal consent was asked for outcome assessment in the first cohort from 2013 to 2016. For a larger clinical study, from 2017, written informed consent was obtained from the legal representative for the EEG measurements and (if possible) from the patient during follow-up.

### Outcome assessment

Patient outcome assessment at 12 months after admission was performed by telephone calls by one of two investigators. The primary outcome measure was the extended Glasgow Outcome Scale (GOSE) approximately after 1 year after TBI dichotomized as poor outcome (GOSE 1–2) or good outcome (GOSE 3–8).

### Clinical parameters

Age, gender, time of TBI, and Injury Severity Score were noted at admission. From the electronic patient records, the following data during the first 7 days of inclusion were retrospectively collected: administration of propofol, fentanyl, midazolam, and mean arterial blood pressure (MAP). Age is a powerful prognostic factor in recovery after TBI [2] and hypotension is associated with poor outcome and increased mortality after TBI [2, 22, 23]. The features age and MAP are available in all patients at any time.

### IMPACT prediction and parameters

For each patient, the prediction by the IMPACT classifier was calculated online [24]. IMPACT parameters are age, motor score, pupil response, CT characteristics (Marshall CT classification [25], epidural hematoma, traumatic subarachnoid hematoma), secondary insults (hypotension and hypoxia), and laboratory parameters (glucose and hemoglobin) at admission, which we retrieved retrospectively from the medical records.

### EEG recordings

Continuous EEG recordings were started as soon as possible after admission to the ICU and continued until 7 days, unless a patient regained consciousness or ICU treatment was ended. Nineteen electrodes (either silver/silver chloride cup or subdermal wire) were placed according to the 10–20 International System. A Neurocenter EEG system with Refa amplifiers (TMSi, Netherlands) was used, recording at a sample frequency of 256 Hz.

### EEG features

At 24, 48, 72, and 96 h after TBI, 60 min of EEG data was preprocessed using a zero-phase sixth-order Butterworth bandpass filter of 0.5–30 Hz. A custom-made computer algorithm was used to detect artifacts and remove the concerning channels within 10-s windows in the common average reference. Artifacts included empty channels and channels with large peaks or noise (amplitude ≥ 150 or ≤ − 150 μV and variance ≥ 1400 or ≤ 1 μV^2^). After preprocessing, all qEEG features were calculated for each 10-min window in the longitudinal bipolar montage.

#### Absolute power per band

The absolute power of the frequency bands was calculated by integration of the power spectral density (PSD) within each frequency band: delta (0.5–4 Hz), theta (4–8 Hz), alpha (8–13 Hz), and beta (13–20 Hz). Hereby, we estimated the PSD of each channel using Welch’s method, with an overlap of 50%, and averaged the PSD over the 60 epochs within the 10-min windows.

#### Total power

The sum of all power bands (0.5–20 Hz) resulted in the total power.

#### Relative power per band

The relative power of each frequency band was defined as the ratio between the power within that frequency band and the total power.

#### ADR

The alpha/delta ratio (ADR) was calculated as the power ratio from the alpha (8–13 Hz) and delta (0.5–4 Hz) frequency bands.

#### SEF90

The spectral edge frequency 90% (SEF90) is the frequency at which 90% of the power lies below in the higher frequencies.

#### Variability per frequency band

Variability in the power of each frequency band was computed by the ratio of the median absolute deviation (MAD) to the median power in each frequency band [9], resulting in a value between 0 (no variability) and 1 (high variability).

#### Brain symmetry index

The pairwise derived brain symmetry index (BSI) was used to calculate the symmetry of power between each pair of electrodes from the left and right hemisphere [26], expressed in a value between 0 (symmetric) and 1 (highly asymmetric). BSI was calculated over the frequency ranges 0.5–20 Hz (total power) and 0.5–4 Hz (delta power).

#### Center of gravity

The center of gravity (COG) quantifies the distribution of power over the head and represents the topographical place at which the maximal power within a certain frequency band. The COG was calculated for the left-right (*x*-) and anterior-posterior (*y*-) direction, expressed as values from − 1 to 1 [27].

#### Mean amplitude

The mean amplitude was defined as the standard deviation of the signal.

#### Coherence

Coherence was defined as the mean of all magnitude-squared coherences between all possible combinations of channels using a Hanning window of 4 s and an overlap of 2 s, resulting in a value between 0 (no synchronization) and 1 (full synchronization).

#### Shannon entropy

The Shannon entropy was calculated as defined by Shannon [28]. Higher entropy indicates a more complex and less predictable system [29, 30].

#### Regularity

Regularity is a measure for the continuity of the EEG pattern based on the variance of the amplitude of the signal. Regularity is normalized between 0 and 1, where a higher value indicates a signal with more regular amplitude [6].

EEG preprocessing and feature calculation were performed using MATLAB R2018a (The MathWorks, Inc., Natick, MA).

### Multifactorial model

We trained a Random Forest classifier, which is a supervised machine learning method that combines multiple individual classification trees using bootstrapped samples to randomly select features at each node to determine a split. Predictions result from the percentage of vote of the different classification trees [31]. Because the prediction accuracy of a model increases by using a larger sample size [32], we used an extended number of observations: for each patient, EEG features from the six 10-min windows at 24, 48, 72, and 96 h after TBI were included. If there was no available data for patients at these intervals, available data from the previous and following hour (and subsequently, the second last or next hour) were used.

Modeling was performed using the Random Forest package in the R Library (randomForest, R 3.5.1) based on Breiman’s Random Forests for Classification [31]. The optimal number of trees was found by observing the out-of-bag error and was set at 100 and the number of maximum nodes was set at 20. As a first step, we trained a Random Forest classifier combining all EEG features, age, and MAP for the prediction of outcome at 24, 48, 72, and 96 h after TBI. Likewise, models were computed on all (six) possible combinations of two intervals. Second, the 10 most important features were selected and used to create new models. Third, new models were created in which the individual parameters from the IMPACT model were added to the models with 10 parameters.

The performance in the training set was evaluated using “leave-one-patient-out.” Poor outcome was defined as positive classification. The area under the ROC curve (AUC) with 50% confidence intervals was used for model comparison. The model with the highest AUC without IMPACT parameters (1) and including IMPACT parameters (2) was used for validation on an external dataset not used for training, and for comparison with the IMPACT predictor (3). Finally, these three models were also trained and evaluated to predict poor outcome defined as GOSE 1–4.

## Results

**Fig. 1:**
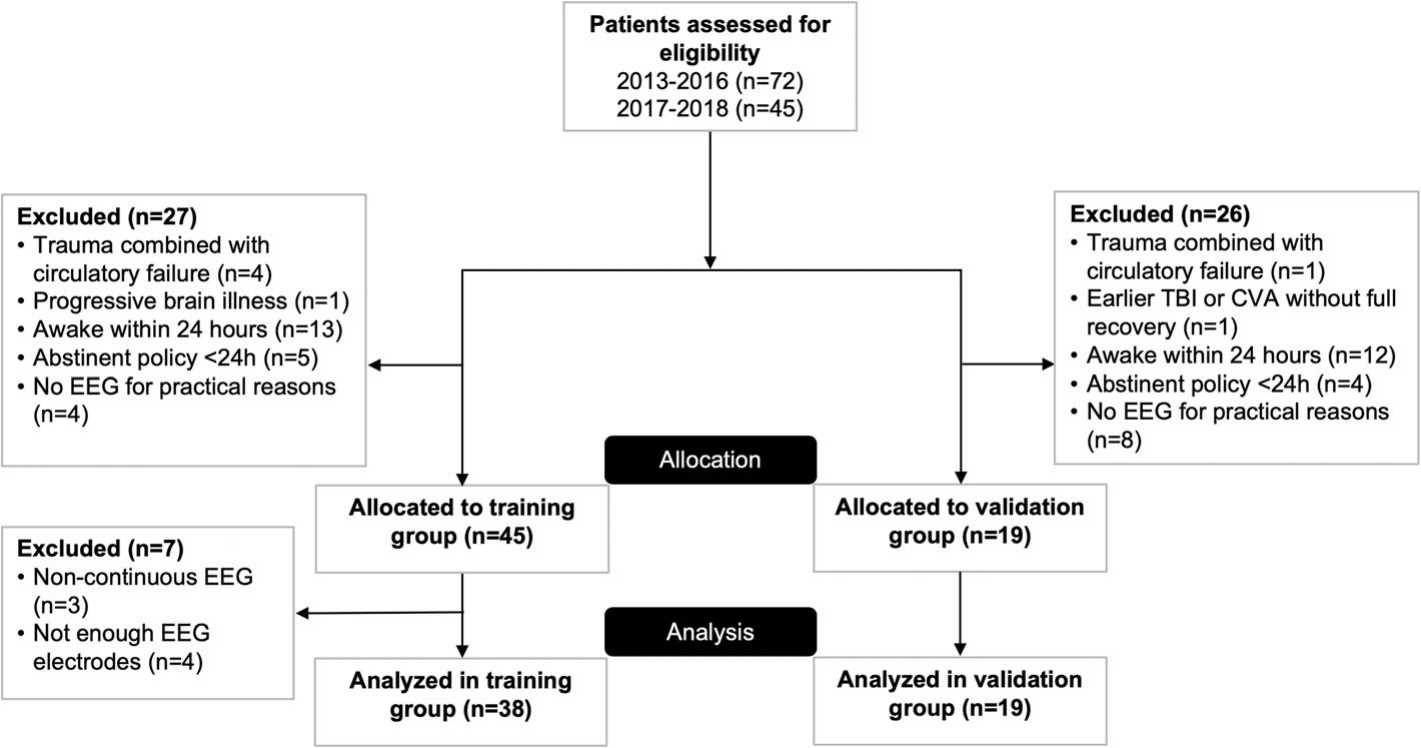
Flow diagram for inclusion and exclusion of eligible patients. Exclusion criteria were trauma following or combined with severe circulatory failure (cardiac arrest/cerebral hemorrhage), earlier TBI or CVA without full recovery, progressive brain illness (tumor, neurodegenerative disease), or limited life expectancy (< 6 months) prior to TBI. Besides, patients were not included because of practical reasons, for example, if the research team was not aware of the admission of a patient

Fifty-seven patients were included. Patients were divided into a training set (38 patients included between 2013 and 2016) and a validation set (19 patients included between 2017 and 2018). A flow diagram for the inclusion and exclusion of eligible patients is shown in Fig. 1.

Table 1 shows the baseline characteristics. Patients with good neurological outcome were significantly younger in both training set (*p* = 0.037) and validation set (*p* = 0.016). Injury Severity Score and duration of EEG monitoring did not differ between the groups in both sets.

**Table 1:** Patient characteristics for both training and validation sets

Descriptive	Training set			Validation set		
	GOSE 1–2 (*n* = 17)	GOSE 3–8 (*n* = 21)	*p* value	GOSE 1–2 (*n* = 8)	GOSE 3–8 (*n* = 11)	*p* value
Gender (m/f (%))	15/2 (88.2)	14/7 (66.7)	–	6/2 (75.0)	9/2 (81.8)	–
Age in years (median (IQR))	58.0 (44.8–70.0)	36.0 (20.0–57.3)	*p* = 0.037*	60.7 (56.3–70.3)	43.8 (33.3–55.2)	*p* = 0.016*
Injury Severity Score (median (IQR))	22.0 (17.5–35.8)	29.0 (26.0–40.2)	*p* = 0.146	27.0 (16.0–35.5)	22.0 (16.5–29.0)	*p* = 0.450
ICU stay in days since trauma (median (IQR))	7.0 (5.0–10.0)	13.0 (9.5–15.2)	*p* = 0.014*	14.7 (11.3–31.9)	22.6 (6.3–30.6)	*p* = 0.904
EEG start in hours after trauma (median (IQR))	15.0 (8.0–19.0)	17.0 (8.0–25.2)	*p* = 0.586	16.8 (9.8–21.9)	4.5 (3.3–7.5)	*p* = 0.033*
EEG recording time in hours (median (IQR))	137.0 (95.8–162.8)	144.0 (98.8–168.0)	*p* = 0.744	163.3 (138.1–166.1)	128.2 (73.0–157.8)	*p* = 0.062
ICP (yes/no (%))	12/5 (70.6)	7/14 (33.3)	–	5/3 (62.5)	2/9 (18.2)	–
Decompressive craniectomy (yes/no (%))	6/11 (35.3)	1/20 (4.8)	–	2/6 (25.0)	2/9 (18.2)	–
Medication administration (yes/no (%))						
Propofol	15/2 (88.2)	19/2 (90.5)	–	8/0 (100)	11/0 (100)	–
Midazolam	10/7 (58.8)	8/13 (38.1)	–	7/1 (87.5)	6/5 (54.5)	–
Fentanyl	15/2 (88.2)	21/0 (100)	–	8/0 (100)	11/0 (100)	–
Noradrenaline	15/2 (88.2)	19/2 (90.5)	–	8/0 (100)	11/0 (100)	–

*GOSE* Extended Glasgow Outcome Scale, *IQR* interquartile range, *ICU* intensive care unit, *EEG* electroencephalography, *ICP* intracranial pressure

*Significant at two-sided Mann-Whitney *U* test with *p* < 0.05

Fifteen out of the 38 patients in the training set died during their hospital admission (GOSE 1), and 15 had a good neurological outcome at 1 year with a GOSE between 3 and 6. The remaining eight patients were lost to follow-up, because they could not be contacted by telephone. Patient outcome was therefore estimated based on their medical data records. Two of them were in a vegetative state at discharge and assigned to the poor outcome group (GOSE 1–2), and the other six to the good outcome group (GOSE 3–8). In the validation set, none of the patients was lost to follow-up; 11 out of 19 patients had a good neurological outcome, and the other eight patients had a poor outcome.

### Model selection and performance in the training set

Our initial models based on all qEEG features, age, and MAP had an AUC of 0.79–0.83. Models in which we selected only the 10 best features had an average AUC of 0.86 on the single time intervals, 0.88 on subsequent combined intervals (24 and 48, 48 and 72, and 72 and 96), and 0.82 on the 1-day-in-between combined intervals (24 and 72, 48 and 96). From these models with fewer features, the best model was the one with features from both 72 and 96 (72 and 96) h after TBI with an AUC of 0.88. Adding IMPACT parameters to the features of our best model resulted in an increase in AUC to 0.94 (specificity 100%, sensitivity 75%) on the training set. This model was subsequently used for validation and compared to the IMPACT predictor independently.

### Performance in the validation set

**Fig. 2:**
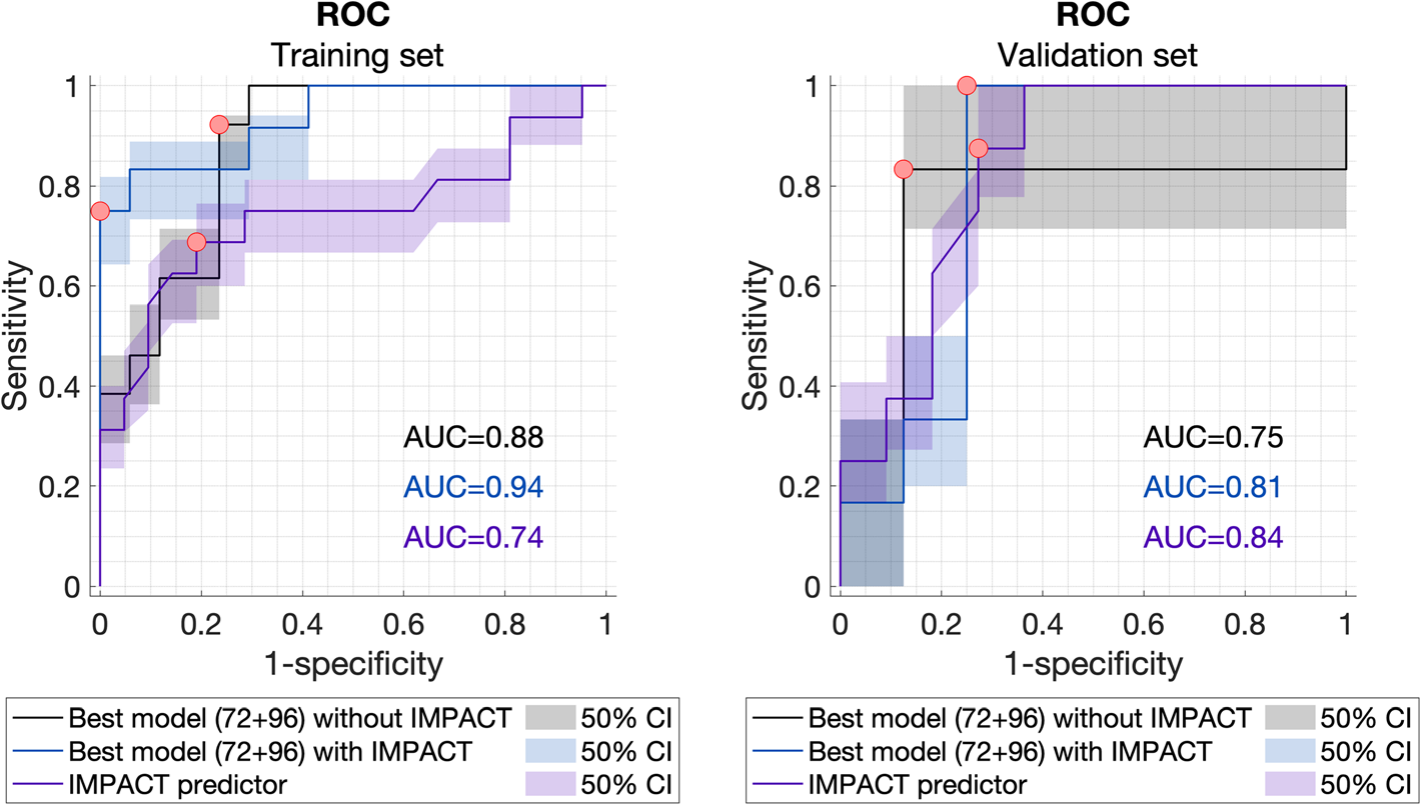
Receiver operating characteristic (ROC) curves with 50% confidence interval of our best models with and without IMPACT features and the online International Mission for Prognosis And Clinical Trial Design (IMPACT) predictions of poor outcome (Extended Glasgow Outcome Scale 1–2) in the training set (38 patients) and validation set (19 patients). The red dots indicate the threshold at which the sensitivity and specificity are best. The area under the curve (AUC) of the model with IMPACT features was higher than our best model without those and similar to the impact predictor alone. The sensitivity and specificity of our best model with IMPACT parameters are slightly higher than those of the IMPACT predictor alone in both the training and the validation sets

Our best model classified poor outcome in the validation set with an AUC of 0.75 without IMPACT parameters and an AUC of 0.81 including IMPACT parameters with a specificity of 83% vs. 75% and a sensitivity of 0.88 vs. 100%, respectively (Fig. 2).

### Performance of the IMPACT predictor

The IMPACT predictor independently predicted with an AUC of 0.74 (sensitivity 65%, specificity 81%) on our training set and an AUC of 0.84 (sensitivity 88%, specificity 73%) on the validation set. However, the sensitivity and specificity at the optimal threshold were lower compared to those of our best model including both EEG and IMPACT parameters (Table 2).

**Table 2:** Model performances of the best model based on electroencephalography features, mean arterial pressure and age, the best model including IMPACT features at admission and the IMPACT predictor independently, predicting poor outcome both as Extended Glasgow Outcome Scale (GOSE) 1–2 and GOSE 1–4. For both the internal validation within the training set and the validation set, the number of patients (*N*), area under the receiver operating characteristic curve (AUC) values with 50% confidence interval, and sensitivity and specificity at an optimal threshold are shown

Performance measures for the prediction model	Best model, 72 + 96 h		Best model, 72 + 96 h + IMPACT		IMPACT predictor	
Poor outcome	GOSE 1–2	GOSE 1–4	GOSE 1–2	GOSE 1–4	GOSE 1–2	GOSE 1–4
Training set						
*N*	30	25	*29*	25	38	30
AUC (50% CI)	0.88 (0.83–0.91)	0.66 (0.57–0.74)	*0.94 (0.89–0.96)*	0.61 (0.51–0.68)	0.74 (0.66–0.79)	0.67 (0.61–0.73)
Threshold	0.541	0.740	*0.680*	0.591	0.650	0.530
Sensitivity	0.92 (0.81–0.98)	0.71 (0.59–0.80)	*0.75 (0.61–0.85)*	0.76 (0.66–0.85)	0.65 (0.54–0.75)	0.63 (0.53–0.72)
Specificity	0.77 (0.66–0.85)	0.75 (0.57–0.88)	*1.00 (0.92–1.00)*	0.50 (0.33–0.67)	0.81 (0.72–0.88)	0.82 (0.67–0.91)
Validation set						
*N*	14	14	*14*	14	19	19
AUC (50% CI)	0.75 (0.60–0.86)	0.73 (0.62–0.82)	*0.81 (0.69–0.88)*	0.76 (0.65–0.89)	0.84 (0.76–0.89)	0.87 (0.78–0.92)
Threshold	0.623	0.805	*0.378*	0.668	0.550	0.349
Sensitivity	0.83 (0.61–0.95)	0.78 (0.61–0.89)	*1.00 (0.79–1.00)*	0.89 (0.73–0.97)	0.88 (0.70–0.96)	1.00 (0.88–1.00)
Specificity	0.88 (0.70–0.96)	0.80 (0.55–0.94)	*0.75 (0.57–0.88)*	0.80 (0.55–0.94)	0.73 (0.58–0.84)	0.75 (0.57–0.88)

*IMPACT* International Mission for Prognosis And Clinical Trial Design, *GOSE* Extended Glasgow Outcome Scale, *AUC* area under the receiver operating characteristic curve, *CI* confidence interval

Performences of the best model referred to throughout the manuscript is shown in italic

Model performances of the best models with and without IMPACT parameters and the performance of the IMPACT predictor are shown in Table 2. This table also shows performances of these models when using an adjusted GOSE dichotomy of 1–4 vs. 5–8.

### Feature contributions

**Fig. 3:**
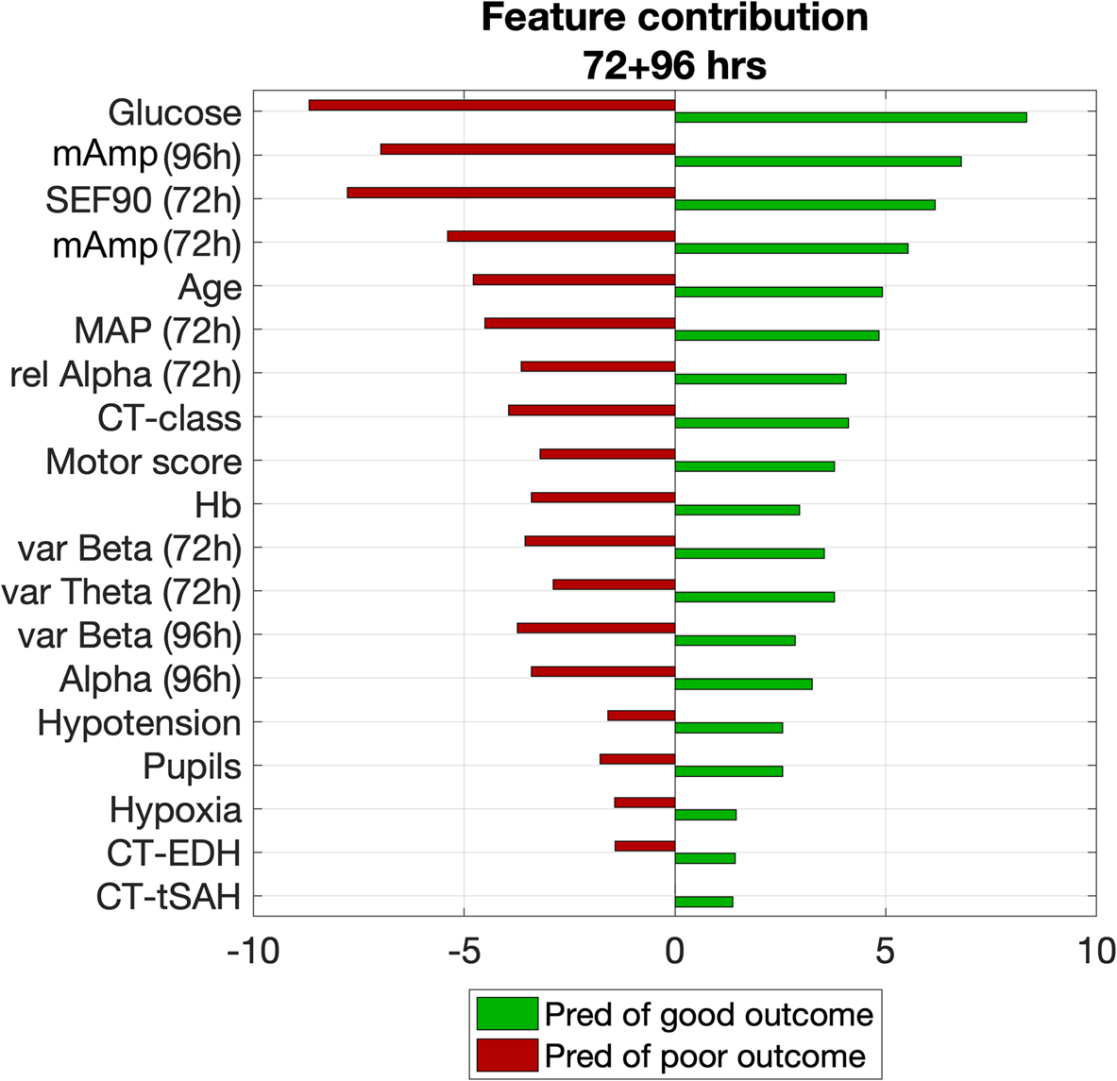
Feature contribution of the best models at 72 + 96 h after traumatic brain injury. Mean amplitude of the electroencephalography (std), age, and mean arterial blood pressure (MAP) were important features. Glucose level at admission strongly contributed to the predictive ability of the models. Pupillary reactivity (pupils), hypotension, hypoxia, and the presence of epidural hematoma or traumatic subdural hemorrhage at the CT scan (CT-EDH and CT-tSAH respectively) were the least relevant features. The bars indicate the contribution of the features in the prediction of good or poor outcome

The best models used 19 features (8 qEEG, MAP, and age, and 9 other IMPACT parameters at admission), of which each contribution is shown in Fig. 3 ranking from high to low relevance. Mean amplitude of the EEG, age, and MAP were important features in all models. In the model at 72 + 96 h after TBI, the most relevant features originated from 72 h, while the mean amplitude at 96 h also contributed strongly to the model’s predictability. From the IMPACT parameters, the glucose level at admission strongly contributed to the predictive ability of the models. Second, motor score and hemoglobin level at admission were features with moderate relevance. The least relevant were pupillary reactivity, hypotension, hypoxia, and the presence of epidural hematoma or traumatic subdural hemorrhage at the CT scan. The latter also scored lower than the EEG features.

## Discussion

We developed a multifactorial model as proof of concept for using a machine learning algorithm including qEEG features and clinically relevant parameters for the prognostication in moderate to severe TBI. Our best models comprised qEEG features and MAP at 72 and 96 h after TBI, combined with clinical (IMPACT) parameters at hospital admission. Our model has an AUC of 0.94 on the training set and 0.81 on the validation set. In both training and validation sets, the sensitivity and specificity of our best model were slightly higher than those of the IMPACT predictor alone, while the AUC of the ROC was similar.

Although several attempts have been made over the last decades to relate individual EEG parameters to patient outcome after TBI [9–11, 13–16, 19, 33–37], no models based on multiple qEEG parameters predicting patient outcome in the acute phase after moderate to severe TBI were reported.

Quantitative EEG features highly contributed to the predictive ability of the model, especially the mean amplitude of the EEG. Although mean amplitude (or total power) of the EEG has been associated with depth of coma [38] and with neurologic outcome in patients after cardiac arrest [6], it has rarely been described in relation to patient outcome after TBI [9]. Spectral edge frequency of 90% and relative alpha power at 72 h were the second and third most relevant qEEG features. This is in line with a recent study from Tolonen et al., who showed that median alpha power was most related to patient outcome after TBI [9].

Age and MAP at 72 h were relevant prognostic features as well, which is in line with earlier literature stating that age is a powerful prognostic factor in recovery after TBI [2]. Prediction was most optimal with features from recordings at 72 and 96 h after TBI. This time interval coincides with being at risk for secondary damage, and this period might therefore have strong impact on the prognosis of TBI patients [39]. Although the model at 72 + 96 h was selected as the best model for this proof of concept, this was however not statistically substantiated and all models performed well at the different (individual) time intervals in the training set (average AUC of 0.86). Therefore, models at other time intervals could be of interest in future studies, too. In addition, temporal evolution might have additional prognostic value. At 72 and 96 h, the number of patients dropped due to patients’ passing or awakening and being transferred to the general ward. Patients with no immediate risk of death and uncertainty about the awakening remain, which is also the category for which caregivers could use better decision support tools.

From the IMPACT parameters, glucose levels at admission contributed most to the model’s predictive ability. Murray et al. already stated that prognostic models in TBI should include laboratory data and especially glucose [2], although they found a smaller effect of glucose compared to other parameters in the IMPACT predictor [24]. Least relevant for our models were pupillary reactivity, hypotension, hypoxia, and the presence of epidural hematoma (EDH) or traumatic subdural hemorrhage at the CT scan. On the contrary, Steyerberg et al. found that most prognostic information was contained by age, motor score, and pupillary reaction and that CT information provided additional information, although this information might be not detailed enough for prognostic purposes [24]. Lee et al. (2019) [40] recently showed that the absence of a posterior dominant rhythm, absence of sleep stage N2 transients, and predominant delta activity and discontinuous background during the first 72 h were individually associated with poor outcome at 3 months and increased the predictive ability of the IMPACT predictor from 0.65 to 0.77. Since we focused on quantitative analysis instead of visual EEG analysis, these EEG characteristics were not incorporated in our proof of concept study; however, they could be added in future prediction modeling after TBI.

EEG measurements are more time consuming than IMPACT prediction. Electrode application takes approximately 45 min, and visual analysis approximately 1–2 h per day. This can be substantially reduced by computer algorithms supporting visual analysis [6] and promising developments in electrode design [41–44]. If the prognostic ability of a model based on EEG exceeds that of the IMPACT predictor, the benefits may outweigh the extra efforts.

A strength of our study is the recording of 7-day high-quality continuous EEG measurements in patients after moderate to severe TBI. Another strength is that in contrast to the IMPACT predictor that only uses parameters at admission, predictions based on longitudinal qEEG parameters take into account the influence of treatment and heterogeneous pathology evolvement in TBI patients [4]. The fact that our best model has similar predictive power as the IMPACT data set independently is an encouraging result for a model that is trained on only 30 patients from a by nature heterogeneous group of TBI patients. A Random Forest classifier was chosen because of its ability to prevent overfitting and the model was trained using leave-one-patient-out, which also lowers overfitting. A limitation of Random Forest models for multifactorial data is that it excludes observations with missing data. For this reason, other possible relevant clinical parameters, which were not available in all patients, such as intracranial pressure, were not considered in modeling.

A limitation of our study is that primary outcome, the GOSE, does not take non-neurological cause of death (in case of polytrauma patients) into account and that patient survival depends on clinical decision-making. In addition, preferably, the poor outcome group would be defined as death to severe disability (GOSE 1–4) and good outcome as moderate disability to good recovery (GOSE 5–8). Due to a low number of patients with a GOSE of 5–8 and incomplete follow-up in 8 patients of the training set, we primarily choose to define poor outcome as GOSE 1–2. Model performances were indeed lower when predicting poor outcome defined as GOSE 1–4. A disadvantage of including the MAP as a parameter for prediction is that it could be artificially controlled because of treatment to optimize hemodynamics or cerebral perfusion pressure. Although MAP was included in the prediction model, its contribution was relatively low (not in the top 5 most relevant factors). As EEG features also depend on the MAP and CPP, this may explain its low additional value for outcome prediction. QEEG features were not corrected for the potential influences of decompressive craniectomy or epileptic activity. Although this might have increased the mean amplitude of the EEG, lower mean amplitude is a strong predictor for poor outcome.

Another limitation of the study is that the training and validation sets originate from different cohorts in time. However, we do not expect this to be a confounder, because there were no changes in treatment over this period and the ratio of poor outcome was relatively similar. The size of the training set and validation set are below the ideal size for a prognostic model, its validation and comparison to the IMPACT predictor alone. However, in this proof of concept study, we showed the potential of Random Forest models for this purpose.

Models should be further developed using larger datasets and use GOSE 1–4 and 5–8 to distinguish between poor and good neurologic outcome respectively. For future research, higher generalizability could be obtained by including more data from multiple time periods and medical centers [9] and by optimizing model parameters (i.e., amount of trees and nodes).

We showed that qEEG parameters provide useful information in prediction of outcome after moderate to severe TBI during the first days after trauma. Ultimately, using data from multiple modalities that monitor brain function might lead to better decision support tools in moderate to severe TBI patients at the ICU.

## Conclusion

Multifactorial Random Forest models using qEEG features, clinical data, and radiological findings have potential to predict neurological outcome in patients with moderate to severe TBI.

## Data Availability

The datasets used and/or analyzed during the current study are available from the corresponding author on reasonable request.

## Abbreviations

ADR: Alpha/delta ratio
AUC: Area under the receiver operating characteristic curve
BSI: Brain symmetry index
cEEG: Continuous electroencephalography
CI: Confidence interval
COG: Center of gravity
CT: Computed tomography
EEG: Electroencephalography
GCS: Glasgow Coma Scale
GOSE: Extended Glasgow Outcome Score
ICU: Intensive care unit
IMPACT: International Mission for Prognosis And Clinical Trial Design
ISS: Injury Severity Score
MAD: Median absolute deviation
MAP: Mean arterial blood pressure
PSD: Power spectral density
qEEG: Quantitative electroencephalography
SEF90: Spectral edge frequency 90%
TBI: Traumatic brain injury
